# Supplementary material for: Assessment of Satisfaction with Pharmacist-Administered COVID-19 Vaccinations in France: PharmaCoVax
Source: Vaccines (Basel). 2022 Mar 14;10(3):440. doi: 10.3390/vaccines10030440 (PMC8950393; doi:10.3390/vaccines10030440)
Supplement: Supplementary file 1 [file vaccines-10-00440-s001.zip › Supplementary Material.pdf]

Supplementary Material—Networks involved in distributing the study

Networks involved in the PharmaCoVax study:

- The Regional Unions of Health Professionals (*Union régionale des professionnels de santé*).
- The French-speaking Society of Medicinal Pharmaceutical Sciences (*Société Francophone des Sciences Pharmaceutiques Officinales*).
- The College of Consultant Pharmacists and Training Supervisors *(Collège des Pharmaciens Conseillers et Maîtres de Stage)*.
- Federation of pharmaceutical unions of France *(Fédération des Syndicats Pharmaceutiques de France* – FSPF, first union).
- Association of pharmacists' unions *(Union des Syndicats de Pharmaciens d'Officine* – USPO, second union).
