# Supplementary material for: Assessment of Satisfaction with Pharmacist-Administered COVID-19 Vaccinations in France: PharmaCoVax
Source: Vaccines (Basel). 2022 Mar 14;10(3):440. doi: 10.3390/vaccines10030440 (PMC8950393; doi:10.3390/vaccines10030440)
Supplement: Supplementary file 1 [file vaccines-10-00440-s001.zip › Supplementary results.pdf]

Supplementary results—Respondent opinion depending on previous influenza vaccination

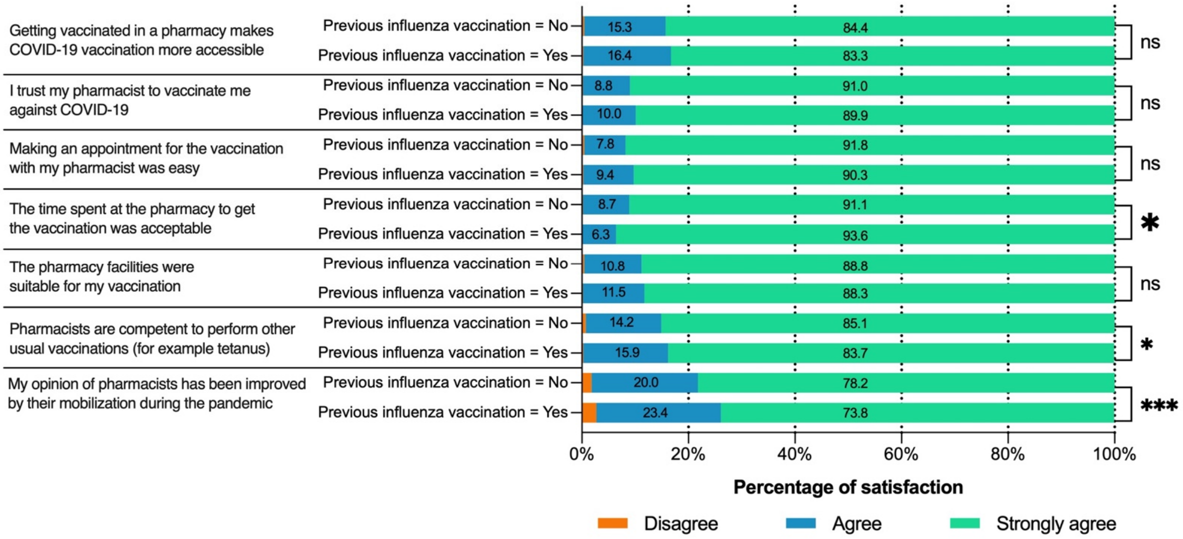

Figure S1. Respondent opinions depending on previous influenza vaccination. Vaccination against influenza is recommended for the most vulnerable individuals (people aged 65 and over, pregnant women, people with certain chronic conditions, and obese people with a Body Mass Index (BMI) greater than or equal to 40) [31].

ns = not significant, * = significant ($p < .05$), ** = very significant ($p < .01$), *** = extremely significant ($p < .001$.
